# Pericyte-specific expression of PDGF beta receptor in mouse models with normal and deficient PDGF beta receptor signaling

**DOI:** 10.1186/1750-1326-5-32

**Published:** 2010-08-25

**Authors:** Ethan A Winkler, Robert D Bell, Berislav V Zlokovic

**Affiliations:** 1 Center for Neurodegenerative and Vascular Brain Disorders, Department of Neurosurgery, University of Rochester, Rochester, NY, USA

## Abstract

**Background:**

Pericytes are integral members of the neurovascular unit. Using mouse models lacking endothelial-secreted platelet derived growth factor-B (PDGF-B) or platelet derived growth factor receptor beta (PDGFRβ) on pericytes, it has been demonstrated that PDGF-B/PDGFRβ interactions mediate pericyte recruitment to the vessel wall in the embryonic brain regulating the development of the cerebral microcirculation and the blood-brain barrier (BBB). Relatively little is known, however, about the roles of PDGF-B/PDGFRβ interactions and pericytes in the adult brain in part due to a lack of adequate and/or properly characterized experimental models. To address whether genetic disruption of PDGFRβ signaling would result in a pericyte-specific insult in adult mice, we studied the pattern and cellular distribution of PDGFRβ expression in the brain in adult control mice and F7 mice that express two hypomorphic *Pdgfrβ *alleles containing seven point mutations in the cytoplasmic domain of PDGFRβ that impair downstream PDGFRβ receptor signaling.

**Results:**

Using dual fluorescent *in situ *hybridization, immunofluorescent staining for different cell types in the neurovascular unit, and a fluorescent *in situ *proximity ligation assay to visualize molecular PDGF-B/PDGFRβ interactions on brain tissue sections, we show for the first time that PDGFRβ is exclusively expressed in pericytes, and not in neurons, astrocytes or endothelial cells, in the adult brain of control 129S1/SvlmJ mice. PDGFRβ co-localized only with well-established pericyte markers such as Chondroitin Sulfate Proteoglycan NG2 and the *xLacZ4 *transgenic reporter. We next confirm pericyte-specific PDGFRβ expression in the brains of F7 mutants and show that these mice are viable in spite of substantial 40-60% reductions in regional pericyte coverage of brain capillaries.

**Conclusions:**

Our data show that PDGFRβ is exclusively expressed in pericytes in the adult 129S1/Sv1mJ and F7 mouse brain. Moreover, our findings suggest that genetic disruption of PDGFRβ signaling results in a pericyte-specific insult in adult F7 mutants and will not exert a primary effect on neurons because PDGFRβ is not expressed in neurons of the adult 129S1/SvlmJ and F7 mouse brain. Therefore, mouse models with normal and deficient PDGFRβ signaling on a 129S1/SvlmJ background may effectively be used to deduce the specific roles of pericytes in maintaining the cerebral microcirculation and BBB integrity in the adult and aging brain as well as during neurodegenerative and brain vascular disorders.

## Background

Pericytes are vascular mural cells embedded within the basement membrane of capillaries originally discovered by Rouget in 1873 [1]. In the central nervous system (CNS), pericytes are widely believed to be integral, multifunctional members of the neurovascular unit at the capillary level [2-5]. Pericytes are seen to ensheathe microvascular endothelial cells forming multiple synapse-like "peg-socket" contacts with adjacent endothelial cells in brain capillaries suggesting the possibility of tightly regulated signaling and functional coupling between these two cell-types [4,6,7]. Although it has been known that brain capillaries have much greater pericyte coverage than peripheral vascular beds, the presence and functional responsibilities of CNS pericytes have largely been neglected until the past two decades [6,7].

Much of the recently gained insight into pericyte biology arose from the analysis of pericyte deficient transgenic mice with disrupted platelet derived growth factor B (PDGF-B)/platelet derived growth factor receptor beta (PDGFRβ) signaling [8-13]. During development of brain capillaries PDGFRβ is exclusively expressed in perivascular pericytes [10,14]. In the embryonic neural tube, endothelial-secreted PDGF-B binds to the PDGFRβ receptor located on the pericyte plasma membrane resulting in dimerization of PDGFRβ, subsequent autophosphorylation of cytoplasmic tyrosine residues and binding of SH2 domain containing proteins which in turn initiate a multitude of signal transduction pathways ultimately stimulating the proliferation, migration, and recruitment of pericytes to the vascular wall of newly formed blood vessels [10,14,15]. Complete knockout of *Pdgfb *or *Pdgfrβ *results in a complete lack of pericytes and embryonic lethality [8,9]. Normal PDGF-B/PDGFRβ interactions and corresponding pericyte recruitment have been demonstrated to play a pivotal role in the regulation of the cerebral microcirculation, including regulating angiogenesis, vascular stability, and integrity of the blood-brain barrier during embryonic development [2,7,16]. Although it has been speculated that brain pericytes might fulfill similar roles in the adult brain, there is limited *in vivo *experimental evidence to support such claims. Therefore, the functional roles of brain pericytes in the adult and aging brain are relatively less well understood in part due to a lack of adequate and/or properly characterized experimental models.

To address whether genetic disruption of PDGFRβ signaling would result in a pericyte-specific insult in adult mice and may therefore be used to study the roles of brain pericytes in the adult and aging brain, we sought to characterize the pattern of expression of PDGFRβ in the adult mouse brain in both control mice and viable F7 mice with two hypomorphic *Pdgfrβ *alleles on a 129S1/SvlmJ genetic background [12]. The F7 mice were generated by seven point mutations in which multiple cytoplasmic tyrosine residues at positions 578, 715, 739, 750, 770 and 1008 were substituted with phenylalanine and the tyrosine at position 1020 was replaced with isoleucine. As a result of these mutations, Src-, Grb2-, PI3K-, RasGAP-, SHP-2 and PLC_γ_-dependent downstream signaling of the PDGFRβ receptor is disrupted. Impaired PDGFRβ downstream signaling in turn results in diminished pericyte recruitment to the vessel wall leading to a 55-75% reduction in the number of pericytes in the embryonic CNS at day E14.5 as previously shown [12]. However, the effects of such alterations in PDGFRβ signaling in the adult mouse brain remain unknown.

Through dual fluorescent *in situ *hybridization (FISH), triple immunofluorescent staining for different cell types in the neurovascular unit, and a fluorescent *in situ *proximity ligation assay (PLA) to visualize molecular PDGF-B/PDGFRβ interactions on brain tissue sections, we show for the first time that PDGFRβ is exclusively expressed in pericytes in several brain regions in the adult control 129S1/SvlmJ mouse model, and confirm a similar pattern of pericyte-specific PDGFRβ expression in the adult brain in F7 mutants on the same genetic background. We demonstrate that F7 mutants are viable, but exhibit substantial pericyte reductions in different brain regions offering a prospective model system appropriate for examining the role of pericytes in the adult and aging brain.

## Results

### PDGFRβ pericyte-specific expression in the adult brain of 129S1/SvlmJ mice and F7 mutants

**Figure 1:**
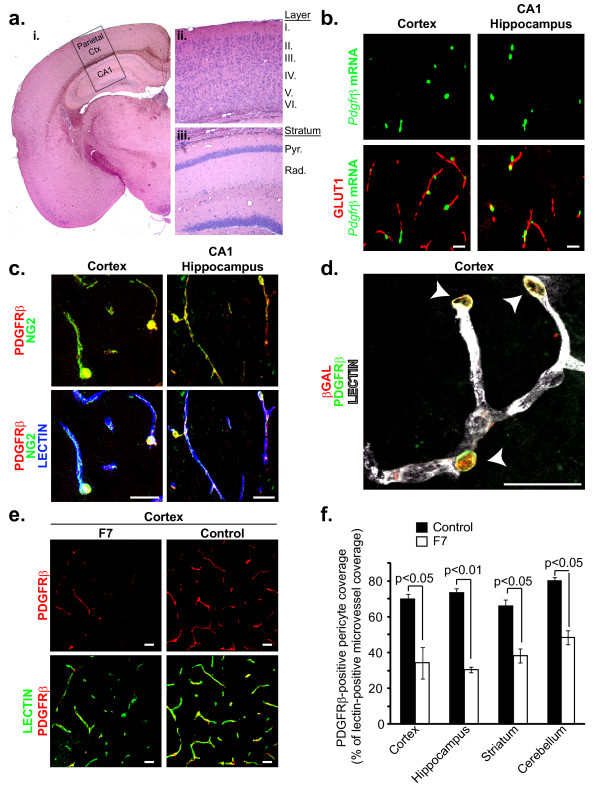
**PDGFRβ expression in perivascular pericytes in the adult brain in control 129S1/SvlmJ mice and F7 mutants with two hypomorphic PDGFRβ alleles**. (**a**) Bright-field imaging analysis of hematoxylin and eosin stained 6 month old 129S1/SvlmJ mouse cerebral hemisphere **i**. Low magnification image depicting brain regions used for fluorescent imaging experiments (broken rectangles). Parietal Ctx, Parietal Cortex; CA1, CA1 hippocampal subfield. **ii-iii**. Higher magnification image depicting the layered cytoarchitecture of the somatosensory parietal cortex (**ii**) and the CA1 hippocampal subfield (**iii**). Roman numerals represent cortical layers. Pyr., Pyramidale; Rad., Radiatum. (**b**) Representative confocal imaging analysis of dual ***in situ ***hybridization for ***Pdgfrβ ***mRNA (green) and endothelial-specific GLUT1 immunodetection in brain capillaries (red) in layer III of the parietal cortex (left) and the stratum pyramidale of the CA1 hippocampal subfield (right) in 6 month old control 129S1/SvlmJ mouse. Scale bar 20 μm. (**c**) Representative three-color confocal imaging analysis of PDGFRβ immunodetection (red), NG2 immunodetection of perivascular pericytes (green), and lectin-positive brain capillaries (blue) in layer III of the parietal cortex (left) and stratum pyramidale of the CA1 hippocampal subfield (right) in a 6 month old 129S1/SvlmJ mouse. Colocalization: yellow. Scale bar 20 μm. (**d**) Representative three-color confocal imaging analysis of PDGFRβ immunodetection (green), β-gal nuclear immunodetection of pericyte-specific LacZ transgene (red), and lectin-positive brain capillaries (white) in layer III of the parietal cortex in a 2 month old x***LacZ4 ***transgenic mouse. Colocalization, arrows: yellow. Scale bar 20 μm. (**e**) Representative confocal imaging analysis of PDGFRβ immunodetection (red) and lectin-positive brain capillaries (green) in the parietal cortex of a 6 month old 129S1/SvlmJ littermate control (right) and F7 mouse (left). (**f**) Graph: PDGFRβ-positive pericyte coverage of lectin-positive brain capillary profiles in the cortex, hippocampus, striatum and cerebellum in 6-8 month old 129S1/SvlmJ controls and F7 mice. Mean ± SEM and n = 6 animals per genotype.

The adult murine cerebrum is comprised of multiple distinct brain structures, including the cortex and hippocampus, each demonstrating a unique cytoarchitecture. In order to provide orientation for later fluorescent images, we first utilized hematoxylin and eosin histological staining to provide a low magnification view of an adult control 129S1/SvlmJ mouse cerebral hemisphere adjacent to sections subsequently utilized for fluorescent imaging experiments (Figure 1a, **panel i**). Further higher magnification analysis demonstrates the distinct layered cytoarchitecture and close approximation between vascular cells, neurons, and glia in the murine parietal cortex (Figure 1a, **panel ii) **and CA1 subfield of the hippocampus (Figure 1a, **panel iii**).

We then determined the expression pattern of *Pdgfrβ *mRNA in adult control 129S1/SvlmJ 6 month old mouse cortex and hippocampus. We show that *Pdgfrβ *mRNA is exclusively expressed in perivascular cells at the capillary level as demonstrated in layer III of the parietal cortex and CA1 subfield of the hippocampus (Figure 1b, **left and right panels**), by performing dual fluorescent *in situ *hybridization combined with endothelial-specific GLUT1 immunostaining. *Pdgfrβ *mRNA expression was restricted to perivascular cells adjacent to GLUT1-positive brain capillaries in frontal and temporal cortices as well as the striatum in the adult 129S1/SvlmJ mouse brain (data not shown).

Next, we studied whether PDGFRβ is an abundant marker expressed in brain pericytes at the protein level that would enable us to quantify pericyte coverage in various brain regions. We show that PDGFRβ is highly expressed in pericytes on lectin-positive brain capillaries as illustrated in both layer III of the parietal cortex and CA1 subfield of the hippocampus (Figure 1c, **left and right panels**) by performing three-color immunofluorescent staining for PDGFRβ and the well-established CNS pericyte marker NG2 [7,17-19] along with endothelial-specific *Lycopersicon Esculentum *lectin fluorescent staining to visualize brain capillaries. To further confirm pericyte-specific expression of PDGFRβ, we utilized the *xLacZ4 *transgenic mouse model which specifically expresses the lacZ nuclear transgenic reporter in committed, differentiated and non-proliferating pericytes/vascular smooth muscle cells [20]. Double immunofluorescent staining demonstrates that PDGFRβ is exclusively expressed in pericytes with β-galactosidase (β-gal) positive nuclear staining located on lectin-positive brain capillaries in both layer III of the parietal cortex (Figure 1d) and CA1 subfield of the hippocampus (data not shown). PDGFRβ expression was also restricted to NG2- or nuclear β-gal-positive pericytes located on lectin-positive brain capillaries in frontal and temporal cortices as well as the striatum in the adult 129S1/SvlmJ mouse brain (data not shown).

As shown in Figure 1c-e, PDGFRβ immunofluorescent staining provides a quantitative fluorescent signal that may be used to determine pericyte coverage of microvascular profiles in mouse brain tissue. Quantification of pericyte coverage in the adult 6-8 month old control 129S1/SvlmJ mouse cortex, hippocampus, striatum and cerebellum revealed a 70, 73, 66, and 80% PDGFRβ-positive pericyte coverage of lectin-positive microvessels, respectively (Figure 1f). Similar analysis of 6-8 month old F7 mutant mice revealed a 34, 30, 38, 48% PDGFRβ-positive pericyte coverage in the cortex, hippocampus, striatum and cerebellum, respectively (Figure 1e-f), indicating a substantial pericyte reduction by 52%, 59%, 43%, and 40%, respectively, compared to controls in different brain regions in this mouse model. The observed reductions in pericyte coverage in the F7 mice were subsequently confirmed in the cortex and hippocampus using desmin, another well-established pericyte marker [14,15,21,22]. Quantification of desmin-positive pericyte coverage in the adult 6-8 month old control 129S1/SvlmJ and F7 mouse cortex and hippocampus revealed a similar 52% and 59% reduction in pericyte coverage of lectin-positive microvessels, respectively.

### PDGFRβ is not expressed in neurons or astrocytes in the adult brain of 129S1/SvlmJ mice and F7 mutants

**Figure 2:**
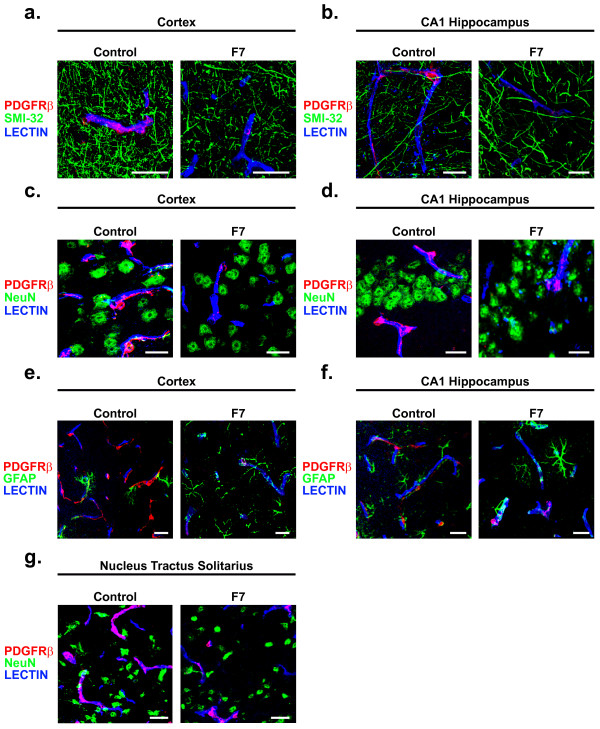
**PDGFRβ is not expressed in neurons or astrocytes in the adult 129S1/SvlmJ and F7 mouse brain**. (**a, b**) Representative confocal imaging analysis of PDGFRβ immunodetection of perivascular pericytes (red), SMI-32 immunodetection of neuronal neurites (green), and lectin-positive brain capillaries (blue) in 6 month old 129S1/SvlmJ control and 6 month old F7 mouse layer II parietal cortex (**a**) and stratum radiatum of the CA1 hippocampal subfield (**b**). Scale bar 20 μm. (**c, d**) Representative confocal imaging analysis of PDGFRβ immunodetection of perivascular pericytes (red), NeuN-specific immunodetection of neurons (green), and lectin-positive brain capillaries (blue) in 6 month old 129S1/SvlmJ control and 6 month old F7 mouse layer III parietal cortex (**c**) and stratum pyramidale of the CA1 hippocampal subfield (**d**). Scale bar 20 μm. (**e, f**) Representative confocal imaging analysis of PDGFRβ immunodetection of perivascular pericytes (red), GFAP-specific immunodetection of astrocytes (green), and lectin-positive brain capillaries (blue) in 6 month old 129S1/SvlmJ control and 6 month old F7 mouse layer III parietal cortex (**e**) and stratum pyramidale of the CA1 hippocampal subfield (**f**). Scale bar 20 μm. (**g**) Representative confocal imaging analysis of PDGFRβ immunodetection of perivascular pericytes (red), NeuN-specific immunodetection of neurons (green), and lectin-positive brain capillaries (blue) in 6 month old 129S1/SvlmJ control and F7 mouse nucleus tractus solitarius in dorsal medulla oblongata. Scale bar 20 μm.

We failed to detect any mouse *Pdgfrβ *mRNA by *in situ *hybridization or PDGFRβ immunostaining by high resolution confocal microscopy in any other cell types in the adult control 129S1/SvlmJ or F7 mouse brain (Figure 1b-e; Figure 2). To confirm that there is no PDGFRβ expression in other cell types in the adult 129S1/SvlmJ and F7 mouse brain, we utilized double immunofluorescent detection coupled to high resolution confocal microscopy imaging of PDGFRβ and either the neurofilament-H marker of neuronal cell processes (SMI-32) (Figure 2a, b), the neuronal-specific nuclear antigen A60 (NeuN) (Figure 2c, d), or the astrocyte-specific glial fibrillary acidic protein (GFAP) (Figure 2e, f) along with endothelial-specific fluorescent lectin staining. We show no detectable colocalization of PDGFRβ immunofluorescent signal with any of the above cell-specific markers in cortex (Figure 2a, c, e), hippocampus (Figure 2b, d, f), or striatum (data not shown) in the adult 129S1/SvlmJ or F7 mouse brain.

In order to investigate whether a similar pericyte-restricted pattern of PDGFRβ expression is also present in non-telencephalic brain structures, we conducted double immunofluorescent detection experiments in the dorsal medulla oblongata including the nucleus tractus solitarius, a nucleus previously reported to have neuronal PDGFRβ expression [23,24]. In contrast to these published results, there was no detectable colocalization of PDGFRβ immunofluorescent signal with NeuN-positive neurons (Figure 2g) or GFAP-positive astrocytes (data not shown).

### PDGF-B/PDGFRβ interactions occur only on perivascular pericytes in the adult mouse brain on 129S1/SvlmJ genetic background

**Figure 3:**
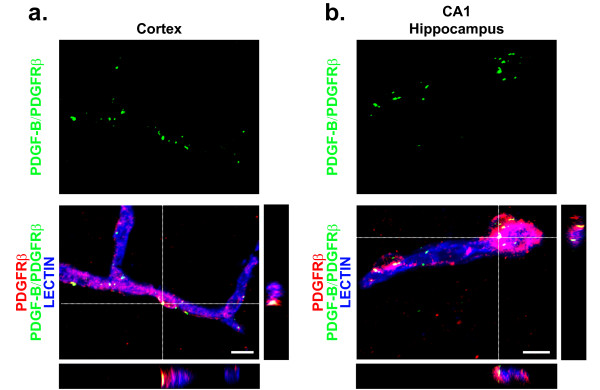
**PDGF-B/PDGFRβ interactions occur exclusively on perivascular pericytes in the adult 129S1/SvlmJ mouse brain**. (**a**) High magnification representative confocal imaging analysis of PDGF-B/PDGFRβ proximity interaction (green), PDGFRβ immunodetection of perivascular pericytes (red), and lectin-positive brain capillaries (blue) in 6 month old 129S1/SvlmJ control mouse layer III parietal cortex. Side panels, orthogonal views. Colocalization: yellow. Scale bar 10 μm. (**b**) High magnification representative confocal imaging analysis of PDGF-B/PDGFRβ proximity interaction (green), PDGFRβ immunodetection of perivascular pericytes (red), and lectin-positive brain capillaries (blue) in 6 month old 129S1/SvlmJ control mouse stratum pyramidale of the CA1 hippocampal subfield. Side panels, orthogonal views. Colocalization: yellow. Scale bar 10 μm.

To further confirm that PDGFRβ is only expressed on brain pericytes and that a lack of PDGFRβ on other CNS cell types is not due to insufficient sensitivity of conventional immunofluorescent staining, we designed a fluorescent *in situ *proximity ligation assay (PLA) to visualize molecular PDGF-B/PDGFRβ interactions in the adult 129S1/SvlmJ mouse brain. PLA is a well-established, antibody based technique designed to visualize endogenous protein-protein interactions with sufficient amplification to detect single molecular events without requiring protein modification, e.g., the creation of fluorescently tagged fusion proteins [25,26]. We show that PDGF-B/PDGFRβ interactions occur only on PDGFRβ-positive pericytes located on lectin-positive brain capillaries in layer III of the parietal cortex (Figure 3a), CA1 subfield of the hippocampus (Figure 3b), and striatum (data not shown) in the adult 129S1/SvlmJ mouse brain.

## Discussion

As our results demonstrate, PDGFRβ is exclusively expressed in brain pericytes and not in neurons, astrocytes or endothelial cells in adult, viable mouse models with normal and deficient PDGFRβ signaling, such as the F7 mutants, on a 129S1/SvlmJ genetic background. Significantly, this pericyte-specific PDGFRβ expression is observed at both the mRNA and protein level in multiple brain regions. Furthermore, PDGFRβ expression is consistently restricted to brain pericytes with use of multiple fixatives and tissue processing techniques during immunofluorescent staining (see **Methods**). Previous studies utilizing mice on a 129Sv/C57BL6 genetic background have also found a similar pericyte-restricted pattern of PDGFRβ expression in mouse brain capillaries during embryonic development [10,14]. Moreover, gene expression profiling on acutely isolated neurons from C57BL6 mice and S100β (S100 calcium-binding protein B) transgenic mice expressing enhanced green fluorescent protein on a hybrid C57BL6/DBA background failed to detect expression of *Pdgfrβ *in neuronal cell populations [27,28].

Intriguingly, recent *in vitro *studies utilizing primary cortical neurons isolated from C57BL6 mice during postnatal day 1 and 6 day old cultures have claimed PDGFRβ expression in cultured neurons after 6 days [29] in contrast to the previous report on acutely isolated non-cultured neurons from C57BL6 mice or mice on C57BL6/DBA genetic background showing a lack of *Pdgfrb *mRNA expression [27,28]. It remains unclear as to whether this discrepancy is the result of alterations in gene expression in response to the growth factors and/or other constituents in the medium during culturing [29], and may therefore not accurately represent the transcriptional profile of neuronal cell populations *in vivo*, as suggested by other studies [30-32] or after an acute isolation of neurons [27,28].

Other studies utilizing adult C57BL6 mice have further claimed that PDGFRβ is expressed in mouse cortical neurons through *ex vivo *immunofluorescent staining and low resolution epi-fluorescent imaging [23,33]. Although these reports demonstrate PDGFRβ labeling consistent with neurons in size, shape and distribution, no colocalization studies were conducted and there is no concluding evidence against or in favor of neuronal PDGFRβ expression. In Ishii et al. 2006 [33], the authors further claim to have deleted PDGFRβ specifically from neurons utilizing a *nestin-Cre *transgenic system and demonstrate no neuronal phenotype but an exacerbated response to cryogenic injury of *nestin-Cre^+^PDGFRβ^flox/flox ^*transgenic mice. The interpretation of Ishii et al. experiments [33] is, however, complicated by recent findings demonstrating that brain pericytes express *nestin *[34] and therefore PDGFRβ expression in pericytes can be affected by *nestin-Cre *genetic manipulation. Since pericyte number and/or coverage have not been determined in the *nestin-Cre^+^PDGFRβ^flox/flox ^*transgenic mouse model [33], it remains uncertain to what extent the observed susceptibility to cryogenic brain injury in this mouse model is due to brain pericyte loss as opposed to primary neuronal insult.

The same group utilizing organotypic brainstem slices from mice on a C57BL6 background in conjunction with single cell patch clamp neuronal recordings has suggested that neuronal PDGFRβ mediates changes in the excitatory postsynaptic currents following application of human recombinant PDGF-BB onto the slices [23,24]. It is of note that PDGF-BB binds to other transmembrane receptors such as platelet derived growth factor receptor alpha (PDGFRα) which is abundantly expressed on neurons in the murine CNS [35-39] and can contribute to the observed changes in postsynaptic currents. As a result, it remains unclear as to whether the observed effects are mediated by binding of recombinant PDGF-BB to neuronal PDGFRα at the concentration utilized and/or whether accompanying PDGF-B/PDGFRβ signaling on brain pericytes may indeed influence neuronal electrophysiological responses further complicating the interpretation of these experiments [24]. In support of our argument, using an *in situ *proximity ligation assay capable of detecting single molecular events, we provide evidence demonstrating that detectable PDGF-B/PDGRβ interactions on tissue sections occurs exclusively on microvascular pericytes following exogenous application of equivalent concentrations (150 ng/mL) of recombinant murine PDGF-BB as in a previous study [24].

The source of the above contradictions between reports demonstrating a lack of neuronal PDGFRβ expression *in vivo *in mouse models on a 129S1/SvlmJ background in the present study and 129SV/C57BL6 background [10,14] and reports suggesting the presence of neuronal PDGFRβ expression in mouse models on a C57BL6 background, including the *nestin-Cre^+^PDGFRβ^flox/flox ^*transgenic mouse model [23,24,33] remains unclear at present time. It is possible, however, that these conflicting results may be due to subtle genetic differences between mouse strains with different genetic backgrounds, as reported by others showing significant strain-specific responses to injury and strain-specific gene expression mapping in the adult mouse brain [40-43]. Intriguingly, previous reports have demonstrated neuronal PDGFRβ expression in the rat CNS [44], whereas our work in progress in the adult human CNS and Alzheimer's disease patients shows a pericyte-restricted pattern of PDGFRβ expression [Winkler EA, Bell RD, Zlokovic BV, unpublished results] indicating that species-specific differences in PDGFRβ expression may indeed exist and that the present mouse models on a 129S1/SvlmJ background could be potentially used as correlates for human studies on brain pericytes.

## Conclusions

Here we have demonstrated that PDGFRβ is exclusively expressed in brain pericytes and may serve as an abundant pericyte-specific marker in the adult 129S1/Sv1mJ and F7 mouse brain. Moreover, these findings suggest that genetic disruption of PDGFRβ signaling in adult, viable mice with this genetic background, such as the F7 mice, will result in a pericyte-specific insult and will not exert a primary effect on neurons because PDGFRβ is not expressed in neurons of the adult 129S1/SvlmJ and F7 mouse brain. Therefore, mouse models with normal and deficient PDGFRβ signaling on a 129S1/SvlmJ genetic background may effectively be used to deduce the specific roles of pericytes in maintaining the cerebral microcirculation and BBB integrity within the neurovascular unit in the adult and aging brain as well as during neurodegenerative and vascular brain disorders.

## Methods

### Animals

Transgenic F7 mutants and their littermate controls on a 129S1/SvlmJ genetic background were provided by Dr. Philippe Soriano (Fred Hutchinson Cancer Res. Cntr., Seattle, WA). The F7 mutants were generated by point mutations in which multiple cytoplasmic tyrosine residues at positions 578, 715, 739 and 750, 770, 1008 were substituted with phenylalanine and the tyrosine at position 1020 was replaced with isoleucine, thereby disrupting Src-, Grb2-, PI3K-, RasGAP-, SHP-2 and PLC_γ_-dependent signal transduction, respectively, as previously described [12]. This results in a substantial pericyte loss in the embryonic CNS, as shown at day E14.5 [12]. *xLacZ4 *mice were provided by Dr. Michelle Tallquist (UT Southwestern, Dallas, TX). Mice were cared for in accordance to the University of Rochester Medical Center Vivarium and Division of Laboratory Animal Medicine guidelines. All procedures were approved by the Institutional Animal Care and Use Committee at the University of Rochester using National Institute of Health guidelines. Mice were anesthetized with a single intraperitoneal injection of ketamine (100 mg/kg) and xylazine (10 mg/kg) and then transcardially perfused with 50 mL PBS containing 5 U/ml heparin. Brain sections were prepared as described below.

### Hematoxylin and Eosin Histologic Staining

This was performed to provide a detailed view of cerebral cytoarchitecture and orientation for fluorescent imaging. Mice were anesthetized, euthanized, and perfused as described above (see Animals). Tissue sections were subsequently fixed overnight in 4% paraformaldehyde (PFA.) Staining was conducted utilizing the FD hematoxylin solution and FD eosin Y solution (FD Neurotechnologies; Catonsville, MD) as described by the manufacturer.

### Digoxigenein (DIG) labeled oligonucleotides

A 129S1/SvlmJ mouse brain cDNA library was created from freshly dissected mouse cortex. A 555 bp fragment of *Pdgfrβ *cDNA was selectively amplified using the following PCR primer combination: 5'- ACCTAGTCGACCACCTTTGTTCTG ACCTGCTC-3' and 5'- TTCGTGGATCCATGGTGATGCTCTCGCCCT-3'. To facilitate directional cloning, primers were designed to include a 5' overhang containing a 5 nucleotide spacer sequence and either a SalI or BamHI restriction site in the forward and reverse primer, respectively. The resulting *Pdgfrβ *amplicon was subsequently ligated into the pBLUESCRIPT II SK vector (Stratagene; Cedar Creek, TX) containing both T3 and T7 RNA polymerase sequences. The plasmid was sent to ACGT Inc. (Wheeling, IL) for sequencing to verify proper insertion. The inserted sequence subsequently aligned to *Mus Musculus Pdgfrβ *mRNA. Both sense and antisense DIG-labeled riboprobes were synthesized with the DIG RNA labeling kit (Roche Applied Science; Indianapolis, IN) utilizing T7 and T3 RNA polymerase, respectively. The DIG-labeled riboprobes were then purified using G-50 sephadex quick spin columns (Roche Applied Science) and diluted to 100 ng/μL in THE RNA storage solution (Ambion; Austin, TX). All riboprobes were aliquoted and stored at -80°C.

### Dual fluorescent *in situ *hybridization

The brains of 6-8 month old 129S1/SvlmJ control mice were studied to determine the pattern of *Pdgfrβ *mRNA expression in the adult brain utilizing dual fluorescent *in situ *hybridization and immunofluorescent staining as previously described [45,46]. Mice were anesthetized and transcardially perfused as described above (see Animals). Snap frozen brains were cryosectioned at 20 μm and stored at -80°C. The following steps were performed at 25°C unless otherwise indicated. All solutions were made using nuclease-free water and glassware was pre-cleaned using RNase Zap reagent (Ambion). In brief, sections were allowed to dry for 30 minutes and then immediately fixed with 4% PFA for 20 minutes. Sections were then incubated with 0.1 M triethanolamine containing 0.25% (v/v) acetic anhydride to minimize non-specific binding of the riboprobe for 10 minutes and were treated with 10 μg/mL proteinase K (Ambion) diluted in PBS for 10 minutes to facilitate tissue penetrance. Sections were then incubated in 5× SSC (0.75 M NaCl, 0.75 M Na-citrate) for 15 min and then prehybridized with prehybridization buffer (5× SSC, 50% formamide, pH to 7.5 with HCl, 100 μg/ml sheared fish sperm DNA) for 2 hours at 65°C. Sections were placed in a humidified chamber and incubated with 300 ng/ml DIG-labeled probe diluted in prehybridization buffer for 12-16 hours at 65°C. Sections were hybridized with either antisense or sense riboprobe as a negative control. Following hybridization, slides were washed with 2× SSC and 0.1× SSC for 1 hour at 70°C. Sections were rinsed with PBS and incubated in 3% H_2_O_2 _for 1 hour to block endogenous peroxidase activity. Tyramide signal amplification (Invitrogen; Carlsbad, CA) was then conducted as described by the manufacturer. In brief, sections were rinsed with PBS and incubated in 10% normal swine serum (Vector Laboratories; Burlingame, CA) for 1 hour. Sections were then incubated with horseradish peroxidase (HRP)-conjugated sheep anti-DIG Fab fragments (Roche Applied Science) for 1 hour and rinsed with PBS. Oregon Green 488-conjugated tyramide diluted in amplification buffer/0.0015% H_2_O_2 _was applied to the sections for 10 minutes. Sections were rinsed with PBS and diluted Oregon Green 488-conjugated tyramide was reapplied for 10 minutes. Sections were then incubated with Alexa Fluor 488-conjugated goat anti-fluorescein/Oregon Green IgG (Invitrogen) overnight at 4°C. To visualize brain endothelial cells, sections were incubated with mouse anti-mouse glucose transporter 1 (GLUT1) IgG (Abcam; Cambridge, MA) overnight at 4°C. To detect GLUT1, sections were incubated with Alexa Fluor 680-conjugated donkey anti-mouse IgG (Invitrogen) for 1 hour.

### Immunodetection of PDGFRβ, Desmin, Chondroitin Sulfate Proteoglycan NG2 and the *xLacZ4 *transgenic reporter

The brains of 6-8 month old 129S1/SvlmJ mice were studied for co-localization of PDGFRβ and the well-established pericyte marker Chondroitin Sulfate Proteoglycan NG2 (NG2) [7,17-19]. Mice were anesthetized and transcardially perfused as described above (see Animals). Snap frozen brain sections were cyrosectioned at 14 μm and subsequently fixed in acetone for 10 minutes at -20°C. Sections were incubated in 10% normal swine serum (Vector Laboratories) for 1 hour at room temperature. Sections were then incubated with goat anti-mouse PDGFRβ IgG (R&D systems; Minneapolis, MN) and rabbit anti-rat NG2 IgG (Millipore; Billerica, MA) which cross-reacts with mouse NG2 overnight at 4°C. For PDGFRβ and NG2 detection sections were incubated with Alexa Fluor 546-conjugated donkey anti-goat IgG (Invitrogen) and Alexa Fluor 488-conjugated donkey anti-rabbit IgG (Invitrogen), respectively, for 1 hour at room temperature. To visualize brain microvascular endothelial cells, sections were incubated with biotin-conjugated *Lycopersicon esculentum *(tomato) lectin (Vector Laboratories) for 1 hour at room temperature followed by incubation with AMCA-conjugated streptavidin (Vector Laboratories) for 30 minutes at 37°C.

To confirm colocalization of PDGFRβ with the *xLacZ4 *transgenic reporter, a marker of committed and differentiated, non-proliferating pericytes/vascular smooth muscle cells [20], snap frozen 2 month old *xLacZ4 *transgenic mouse brain sections were cryosectioned at 14 μm and subsequently fixed in a 1:1 solution of acetone and methanol for 10 minutes at room temperature. Sections were blocked and incubated with goat anti- mouse PDGFRβ IgG (R&D systems) and mouse anti-β-galactosidase IgG (Cell Signaling; Danvers, MA) as described above. For PDGFRβ and LacZ detection sections were incubated with Alexa Fluor 488-conjugated donkey anti-goat IgG (Invitrogen; Carlsbad, CA) and Alexa Fluor 546-conjugated donkey anti-mouse IgG (Invitrogen), respectively, as described above. Brain microvascular endothelial cells were visualized with fluorescent lectin staining as described above.

To study PDGFRβ-positive or desmin-positive pericyte coverage, tissue sections were prepared as described above. Sections were then incubated with goat anti-mouse PDGFRβ IgG (R&D systems) or mouse anti-human desmin IgG (Dako USA; Carpinteria, CA) which cross-reacts with mouse desmin overnight at 4°C. For PDGFRβ and desmin detection sections were incubated with Alexa Fluor 546-conjugated donkey anti-goat IgG (Invitrogen; Carlsbad, CA) or Alexa Fluor 546-conjugated donkey anti-mouse IgG (Invitrogen), respectively, for 1 hour at room temperature. Brain microvascular endothelial cells were visualized with fluorescent lectin staining as described above.

### Immunodetection of neuronal-specific and astrocyte-specific markers

The brains of 6-8 month old 129S1/SvlmJ littermate controls and 6-8 month old F7 mice were studied for colocalization of PDGFRβ with neuronal neurofilament-H (SMI-32), neuronal nuclear antigen A60 (NeuN), or astrocyte glial fibrillary acidic protein (GFAP). Mice were anesthetized as described above and transcardially perfused with 10 mL PBS containing 5 U/ml heparin followed by 30 mL 4% PFA. Brains were carefully removed from the skull and immersion fixed in 4% PFA overnight at 4°C. Forty μm vibratome-sectioned brain sections were incubated in target antigen retrieval solution, pH 9 (Dako USA) for 15 minutes in a 80°C water bath and then blocked and incubated with the following primary antibodies as described above: goat anti-mouse PDGFRβ (R&D systems), mouse anti-mouse SMI-32 (Abcam), mouse anti-mouse NeuN (Millipore), and mouse anti-mouse GFAP (Cell Signaling). PDGFRβ was detected as described above. To detect NeuN, SMI-32, and GFAP sections were incubated with Alexa Fluor 488-conjugated donkey anti-mouse IgG for 1 hour at room temperature. Brain microvascular endothelial cells were visualized with fluorescent lectin staining as described above.

### Quantification of PDGFRβ-positive and Desmin-positive pericyte coverage of lectin-positive brain microvessels

All images were acquired by a blinded investigator using a custom-built Zeiss LSM 510 meta confocal laser scanning microscope (see below) using a Zeiss LD LCI Plan-Apochromat 25×/0.8 Imm Korr DIC water immersion objective. In each mouse, four different 200 μm × 200 μm fields were analyzed per brain region in six nonadjacent sections approximately 100 μm apart. PDGFRβ-postive or desmin-positive immunofluorescent and lectin-positive fluorescent signals from brain microvessels < 6 μm in diameter were individually subjected to threshold processing and the areas occupied by their respective signals were quantified by a blinded investigator using the NIH Image J software Area measurement tool. The percent of PDGFRβ pericyte coverage of lectin-positive microvessels was then deteremined by normalizing the PDGFRβ-positive signal to the lectin positive-brain microvessel signal. We used Graph-Pad Prism software for statistical calculations. One-way analysis of variance followed by a Tukey posthoc test were used to determine statistically significant differences in PDGFRβ-positive pericyte coverage amongst different brain regions in both 129S1/SvlmJ mice and F7 mice. Mean values are reported plus or minus the standard error of the mean (SEM).

### Fluorescent *in situ *PDGF-B/PDGFRβ proximity ligation assay

The brains of 6-8 month old 129S1/SvlmJ mice were studied to determine the localization of PDGF-B/PDGFRβ interactions *in situ*. Mice were anesthetized and transcardially perfused as described above (see Animals). Frozen brain sections were cryosectioned at 14 μm. Sections were rehydrated with PBS and blocked with 10% normal swine serum (Vector Laboratories) for 1 hour at room temperature. Sections were then incubated with 150 ng/mL mouse recombinant PDGF-BB (GenWay Biotech, Inc.; San Diego, CA) for 1 hour at room temperature. Sections were washed with PBS containing 0.05% triton X-100 and subsequently fixed with 4% PFA for 10 minutes at room temperature. Sections were incubated with goat anti-mouse PDGFRβ IgG (R&D systems) and rabbit anti-human PDGF-B IgG (Thermo Scientific; Rockford, IL) which cross reacts with mouse PDGF-B overnight at 4°C. Proximity ligation was then conducted *in situ *as described by the manufacturer (Olink Bioscience; Uppsala, SE) utilizing the Duolink II PLA probe anti-goat PLUS, Duolink II PLA probe anti-rabbit MINUS, and Duolink II detection reagents orange to visualize PDGF-B/PDGFRβ interactions. Following serial SSC washes, sections were rinsed with PBS were incubated with biotin-conjugated *Lycopersicon esculentum *lectin (Vector Laboratories) overnight at 4°C. To visualize brain microvascular endothelial cells and PDGFRβ, sections were incubated with Dylight 649-conjugated streptavidin (Vector Laboratories) and Alexa Fluor 488-conguated donkey anti-goat IgG (Invitrogen), respectively, for 1 hour at room temperature.

### Laser scanning confocal microscopy imaging

All images were acquired using a custom-built Zeiss LSM 510 meta confocal laser scanning microscope with a Zeiss LD LCI Plan-Apochromat 25×/0.8 Imm Korr DIC, C-Apochromat 40X or 63X water immersion objective (Carl Zeiss Microimaging Inc.; Thronwood, NY). A 488 nm argon laser was used to excite Alexa Fluor 488 and the emission was collected through a 500-550 band-pass filter. A 543 HeNe laser was used to excite Alexa Fluor 546 and the emission was collected through a 560-615 band-pass filter. A 633 HeNe laser was used to excite Alexa Fluor 680 and Dylight 649 and the emission was collected through a 650-710 band-pass filter. An 800 nm mode-locked femtosecond pulsed DeepSee Ti:sapphire laser (Spectra Physics; Santa Clara, CA) was used for AMCA excitation and emission was collected using 405-450 nm band-pass filter.

## Competing interests

The authors declare that they have no competing interests.

## Authors' contributions

EAW conducted fluorescent *in situ *hybridization and proximity ligation experiments and helped to draft the manuscript. RBD conducted all immunostaining experiments, quantified pericyte coverage, and helped to draft the manuscript. BVZ designed and supervised all experiments and wrote the manuscript. All authors read and approved the final manuscript.
